# Determination of Toxic Tropane Alkaloids in Globally Sourced Soya, Cereals and Products Using Dilute-and-Shoot Technique Coupled with LC-MS/MS

**DOI:** 10.1007/s12403-025-00718-z

**Published:** 2025-07-01

**Authors:** Rufielyn S. Gravador, Brett Greer, Simon A. Haughey, M. Mar Aparicio-Muriana, Wilfred A. Abia, Alexandra-Irina Mavrochefalos, Anne P. Nugent, Christopher T. Elliott

**Affiliations:** 1 Institute for Global Food Security, School of Biological Sciences, Queen’s University Belfast, Belfast, UK; 2 International Joint Research Center on Food Security (IJC-FOODSEC), 113 Thailand Science Park, 16 Phahonyothin Road, Khong Luang 12120, Pathum Thani, Thailand; 3 Department of Biochemistry (Laboratory of Pharmacology and Toxicology), Faculty of Science, University of Yaoundé 1, BP 812, Yaoundé, Cameroon

**Keywords:** Matrix effect, Plant toxins, Atropine, Scopolamine, Dilute-and-shoot, Solid-liquid extraction

## Abstract

For the first time, this study reports the content of tropane alkaloids (TAs) in soybean and soymeal samples sourced from 13 different countries, as well as in various cereals (including corn, millet, wheat, and sorghum) and a product (Super Cereals). The analysis was conducted using a fully validated solid-liquid extraction (SLE) with dilute-and-shoot (DnS) method along with a liquid chromatography-tandem mass spectrometry (LC-MS/MS) triple quadrupole system. The samples were extracted using a solution of acetonitrile and water (60:40) with 1% formic acid. The limits of quantification (LOQ) were 0.10 µg kg^−1^ and 0.25 µg kg^−1^ for atropine and scopolamine, respectively. The extraction recovery was at least 80%, and the intra- and inter-day precision (relative standard deviation) were below 15% at 1, 5, and 50 µg kg^−1^ atropine and scopolamine spiked levels. The validation parameters showed that the method was fit for the purpose of accurate LC-MS/MS quantification of atropine and scopolamine. The SLE with DnS is high-throughput, enabling the extraction of TAs in 2.3 min per sample or at least 60 samples in 140 min. Whereas typical TA extraction methods require lengthy and costly clean-up and concentration steps, the DnS does not require these steps, sophisticated equipment, or an expert operator for execution, which makes it a simpler and straightforward procedure for TA analysis in different grain-based food matrices. Application of the LC-MS/MS method showed that one in 80 millet or corn, 18 in 269 soybeans, 20 in 62 soymeal and 20 in 42 Super Cereals samples had atropine and scopolamine above the maximum level of 1 μg kg^−1^ set by the EU Commission Regulation. TA contamination in these samples could either be due to the mixing with (visible) or exposure to (invisible) TA-producing plant material. The latter was proven through an exposure experiment, which showed that atropine and scopolamine from *Datura stramonium* seeds can be transferred to food grains (soybeans) invisibly with prolonged contact.

**Supplementary Information:**

The online version contains supplementary material available at 10.1007/s12403-025-00718-z.

## Highlights

- An LC-MS/MS method for the quantification of tropane alkaloids (TAs) was validated.
- Solid-liquid extraction with dilute-and-shoot resulted in clean extracts.
- The extraction procedure was high-throughput and straightforward.
- The method was fit for purpose based on validation parameters.
- The method is suitable for TA analysis in various food grains and products.

**Supplementary Information:**

The online version contains supplementary material available at 10.1007/s12403-025-00718-z.

## Introduction

Tropane alkaloids (TAs) are secondary plant metabolites naturally produced by some plant families as defence mechanisms against diseases, grazing, and insect damage (Adamse et al. 2014; de Nijs et al. 2023). The most well-known TAs, which are also present in the highest concentrations in their plant sources, are atropine (a racemic mixture of (−)-hyoscyamine and (+)-hyoscyamine) and (−)-scopolamine. Of these TAs, only the (−)-hyoscyamine and (−)-scopolamine are produced in TA synthesis (EU 2021), and will be referred to in the current study as atropine and scopolamine.

TA-producing plants such as *Datura stramonium* (thorn apple or Jimson weed), which belongs to the *Solanaceae* family, grow among food crops (e.g., vegetables, and grains such as soybeans and millet) and contaminate foods through accidental co-harvesting or processing (Abia et al. 2021; Adamse et al. 2014; Chan 2017; Stratton 2017). Atropine and scopolamine have anticholinergic or antimuscarinic effects, as they can compete with acetylcholine for binding sites on the muscarinic receptors in the central and autonomic nervous system, affecting heart rate, respiration, and central nervous system functions (Adamse et al. 2014; Chan 2017; Kohnen-Johannsen and Kayser 2019). Several food poisoning incidents have been recorded as a result of the consumption of foods contaminated by atropine and scopolamine or their plant sources (Adamse et al. 2014; Gravador et al. 2024). The Commission Regulation (EU 2023) 2023/915 of 25 April 2023 set the maximum levels, among others, of (1) 1 μg kg^−1^ for atropine and 1 μg kg^−1^ for scopolamine in baby food and processed cereal-based food for infants and young children containing millet, sorghum, buckwheat, corn or their derived products; (2) 5.0 μg kg^−1^ sum of atropine and scopolamine in unprocessed millet and sorghum, and for millet, sorghum, and corn placed on the market for final consumers; and (3) 15 μg kg^−1^ sum of atropine and scopolamine for unprocessed corn grains. In addition, international guidance on the levels of atropine and scopolamine in Super Cereals was recommended by the Joint FAO/WHO Expert Meeting in 2020 based on an intake of 100 g day^−1^ by adults (30 μg kg^−1^ up to 90 μg kg^−1^ in situations like food insecurity). Super Cereal is a nutrition and humanitarian aid that assists approximately 4.9 million people globally with high nutrient needs every year in developing countries (Abia et al. 2021; FAO/WHO 2020; Haughey et al. 2021). It consists of fortified flour of corn or wheat and soy—it is prepared from heat-treated corn and de-hulled soya beans, sugar, dried skim milk, and refined soya bean oil.

Studying the occurrence and levels of atropine and scopolamine in the food supply chain is crucial to protecting consumers’ safety, implementing appropriate regulations, and developing strategies to minimise their presence in foods. In recent years, analytical methodologies have been designed to determine TAs, specifically atropine and scopolamine, alone or with other food contaminants. Chromatographic techniques (gas, thin-layer or liquid), capillary electrophoresis (Dräger 2002; González-Gómez et al. 2022; Romera-Torres et al. 2018a), immunoassays (Xiao et al. 2021) and spectrophotometry (Mardare et al. 2022) have been implemented to study TAs in various matrices. Nevertheless, liquid chromatography-tandem mass spectrometry (LC-MS/MS) has been the most commonly employed technique due to its high selectivity, sensitivity, robustness, and multi-analyte capability.

In TA analysis using LC-MS/MS, sample preparation is often the most challenging step due to the complexity of many different food and feed matrices and the generally low concentrations of analytes. Solid-liquid extraction (SLE) with acidified (using formic or acetic acid) aqueous organic solution (methanol or acetonitrile) is typically used as a solvent for TA extraction in food grains, with optional clean-up steps to obtain higher sensitivity and lower quantification limits (LOQ). Marín-Sáez et al. (2017) obtained lower LOQs for atropine and scopolamine through the use of solid-phase extraction (SPE) (1–2 μg kg^−1^) *vs* without SPE (2–5 μg kg^−1^) when analysing together with other analytes in cereals using LC-MS. Romera-Torres et al. (2018b) also employed SPE with an additional clean-up step using chitosan for LC analysis of TAs and other compounds from feeds, but the LOQs were higher (5–25 μg kg^−1^). On the other hand, Vuković et al. (2022) applied the QuEChERS (quick, easy, cheap, effective, rugged, and safe) method to extract atropine and scopolamine in corn and a low LOQ of 1 μg kg^−1^ for both analytes was achieved, meeting the maximum limit set by the EU for cereal-based foods for infants and young children. Some researchers did not apply clean-up steps but also obtained low LOQ levels for atropine and scopolamine. For instance, Veršilovskis et al. (2020) passed the supernatant (extracts of bread) into a 30 kDa ultrafilter and were able to obtain LOQs of 1 μg kg^−1^ for atropine and 0.8 μg kg^−1^ for scopolamine. In addition, Begemann et al. (2021) only filtered the supernatant (1:10 sample to the solvent ratio of millet) before LC-MS analysis, resulting in LOQs of 0.01 and 0.025 ng ml^−1^ for atropine and scopolamine, respectively.

Another simple sample preparation method that can result in acceptable recovery and matrix effect of multiple analytes is the dilute-and-shoot (DnS) procedure. In DnS, the sample or extract is diluted before filtration and injection for LC-MS/MS analysis, which, due to the dilution of the matrix and target analytes, can lead to higher detection limits (Greer et al. 2021). DnS is a simple, cost-effective, high-throughput, and rapid method that can be applied to TA extraction. The simplicity of DnS lies in the reduced analytical steps in sample preparation and the large number of samples that can be extracted simultaneously, making it attractive, especially for routine analysis involving large numbers of samples (Greer et al. 2021) . Consequently, in this study, an SLE coupled with a DnS procedure to extract atropine and scopolamine in soybeans was optimised and validated for their quantification using an ultra-high-pressure LC-MS/MS. The optimised SLE with the DnS-LC-MS/MS method was further validated and used to determine atropine and scopolamine in other commodities prone to TA contamination, such as cereals. The method was finally applied to determine the contents of atropine and scopolamine in a global (13 countries) collection of soybeans and soymeal, cereals (corn and millet), and Super Cereals. Lastly, the transfer of TA from *D. stramonium* seeds to soybeans was investigated to provide insights into the observed contamination patterns.

## Materials and Methods

### Chemicals and Reagents

Commercial standards of atropine sulphate and scopolamine hydrobromide, and acetic acid were purchased from Sigma-Aldrich (St. Louis, MO, USA). LC-MS grade methanol, acetonitrile, and formic acid were purchased from Honeywell (London, UK and Seelze, Germany). Ultrapure water (resistivity of 18.2 MΩ-cm) was obtained from a Milli-Q system (Millipore, Burlington, MA, USA).

Stock solutions of atropine and scopolamine were individually prepared by accurately weighing 10 mg of each and dissolving in 1 mL Milli-Q water, obtaining a concentration of 10.0 mg mL^−1^ (10 000 ppm). These were diluted with Milli-Q water to attain a working standard at 1 ppm (1000 ppb or µg L^−1^), with the working standard used to prepare the calibration curve by serial dilution, to obtain a 10-point calibration curve from 0.10–100 µg L^−1^ (ppb). All standard solutions were kept in amber glass vials at – 20 °C and brought to room temperature before use. The stock solutions were freshly prepared every two months.

### Sample Extraction Procedure

The extraction method using DnS, employed by Meneely et al. (2023) in extracting mycotoxins in oats, was adopted with some modifications to optimise the extraction of TAs in the samples. Soy (soybean and soymeal) samples from 13 different countries were received at the Institute for Global Food Security (IGFS), Queen’s University Belfast (QUB), United Kingdom (UK). These samples (*n* = 331; Supplementary Table 1-Sample origin) were ground to homogenise and increase surface area for extraction (Cuisinart coffee grinder, Hampshire, UK) and packed in airtight resealable bags before storage at – 20 °C until analysis. Other samples [cooked (*n* = 40) and uncooked (*n* = 40) millet or corn; Super Cereals (*n* = 42)] were received in powdered form, ready for extraction and analysis.

A 1.00 g portion of each ground sample was weighed into a 15 mL polypropylene tube (Sarstedt, Leicester, UK). A 5 mL extraction solution of acetonitrile: water (60: 40, v/v) with 1% formic acid was added and mixed using a vortex before being placed on a multi-tube vortex (Fisherbrand, UK) at 2500 rpm for 90 min, followed by incubation in an ultrasonic bath (CamSonix, UK) for a further 30 min. After centrifugation (Rotina Hettich Zentrifugen, Germany) at 2516 RCF (g Force) for 15 min at 8 °C, an aliquot of 500 µL was diluted 1:1 (v/v) with 500 µL acetonitrile: water (40: 60, v/v) with 1% formic acid in a 1.5 mL Eppendorf tube and mixed briefly in a vortex, before filtration through a 0.2 µm PTFE syringe filter (Merck, Darmstadt, Germany) into an amber vial and finally injected into the LC-MS/MS system.

### Instrumental Analysis

Analysis was performed on a SCIEX ExionLC™ AD system with detection *via* SCIEX triple Quad 5500+ QTrap Ready LC-MS/MS system equipped with Turbo V™ ionisation source (SCIEX, MA, USA). Chromatographic separation was achieved using a Gemini C18 column (100 × 4.6 mm, 5 μm particle size, 110 Å; Phenomenex, Macclesfield, UK) with a temperature of 30 ℃. Both mobile phases contained 0.1% formic acid (v/v) and were composed of water (mobile phase A: MPA) and methanol (mobile phase B: MPB). Elution was carried out in a gradient mode outlined in Table 1, with the flow rate set at 0.80 mL min^−1^. The total run time was 8.5 min, with the injection volume set at 2.5 µL. The mass spectrometer (MS) was operated in positive electrospray ionisation mode (ESI+) with a capillary voltage of 4.5 kV. After every 10 samples, a solvent blank was injected, while after every 20 samples, a solvent blank followed by quality control samples and solvent blank were injected to monitor instrument performance.

**Table 1:** Chromatography gradient elution conditions

Time (min)	MPA (%)	MPB (%)
0	95	5
0.5	95	5
2.5	45	55
4.5	1	99
6.5	1	99
7.0	95	5
8.5	95	5

*MPA* Mobile phase A, *MPB* Mobile phase B

The MS conditions were optimised by infusion of individual standard solutions of atropine and scopolamine at 100 µg L^−1^ in acetonitrile: water (50:50, v/v) to determine the optimum transitions. The precursor and two product ions were monitored in multiple reaction monitoring mode (MRM) for accurate confirmation for each analyte, with their respective analyte-dependent operating conditions outlined in Table 2. The following setting of the ion source was employed: curtain gas = 35, collision gas = 9, ion spray voltage = 4500 V, temperature = 600 °C, ion source gas 1 = 70 and ion source gas 2 = 60. The target scan time was 0.5 s, the MRM detection window was 40 s.

**Table 2:** Optimised MS/MS parameters for the tropane alkaloids quantified

Analyte	Precursor ion (m/z)	Product ion (m/z)	Retention time (min)	DP (V)	CE (V)	CXP (V)
Scopolamine	304.2	138.1 (Q)	2.75	100	27	16
	304.2	156.1 (q)	2.75	100	21	16
Atropine	290.2	124.1 (Q)	3.00	100	31	14
	290.2	93.1 (q)	3.00	100	37	10

*DP* Declustering potential, *CE* Collision energy, *CXP* Collision cell exit potential, *V* volts, *Q* Quantifier ion, *q* Qualifier ion

Analyst^®^ Software 1.7.1 and SCIEX OS-Q Software were used to acquire and process the data. For confirmation of compound identification, the ion ratio has to agree with the related values of the standards within 20%, as stated. For the retention time, a stricter in-house criterion of ± 0.03 min was applied.

### Method Validation

The method validation procedure was adopted from Marín-Sáez et al. (2017) based on SANTE/11312/2021 validation guidelines (EURL 2021) or the Eurachem Guide (Magnusson 2014). To determine extraction recovery (% or efficiency of the extraction), homogenised blank samples were spiked in five replicates with the relevant volume of the working standard to attain the following final concentration levels: 1, 5, and 50 µg kg^−1^. Spiked samples (pre-extraction) were left overnight at room temperature to allow the TAs to interact with matrix components. The spiked concentrations were chosen to ensure that the validation was performed at the relevant levels and that the method was fit for the purpose of quantifying at the maximum levels permitted given in the Commission Regulation (EU 2023) 2023/915 (Section “Introduction”: between 1 and 15 µg kg^−1^ depending on food grain commodities). In addition, based on SANTE/11312/2021, the recovery of an analyte should normally be determined by spiking within a range corresponding to the reporting limit (RL) and 2–10× the RL or at the maximum residue level. Blank sample extracts were spiked post-extraction in quintuplicate with an appropriate volume of the working standard solution to attain the following final concentration levels: 1, 5, and 50 µg kg^−1^ (post-extraction). The peak area of samples spiked pre-extraction and post-extraction were compared to calculate the extraction efficiency (a measure of trueness; Eq. 1).

1 $${\text{Extraction }}\,{\text{recovery }}\,\left( {\text{\% }} \right) = { }\left( {\frac{{{\text{ area }}\,{\text{of}}\,{\text{ sample}}_{{{\text{pre}} - {\text{extraction}}}} }}{{{\text{area }}\,{\text{of}}\,{\text{ sample }}_{{{\text{post}} - {\text{ extraction}}}} { }}}} \right) \times { }100$$

Method precision was tested at the same concentration levels as the extraction recovery (1, 5, and 50 µg kg^−1^). It was performed by repeating the pre-extraction spikes in five replicates (repeatability, intra-day precision). The procedure was repeated across three separate days to determine reproducibility (inter-day precision). The repeatability and reproducibility were expressed as relative standard deviation (RSD, %).

Linearity and matrix effects (signal suppression or enhancement, SSE) were assessed using a matrix-matched calibration, in which blank sample extracts (matrix) were spiked with the working standard containing both atropine and scopolamine to achieve a 10-point calibration curve in the range of 0.10–100 µg kg^−1^. A solvent (external) calibration curve was also prepared by serially diluting the working standard to achieve the same concentrations as in the matrix-matched calibration. Matrix effects (SSE) were then calculated by comparing the slopes of the matrix-matched and solvent calibration curves using Eq. (2).

2 $${\text{Matrix }}\,{\text{effects }}\left( {\text{\% }} \right) = { }\left[ {\left( {\frac{{{\text{slope }}\,{\text{matrix}}}}{{{\text{slope }}\,{\text{solvent}}}}} \right) - 1} \right]{ } \times { }100$$

To estimate the limit of detection (LOD) and LOQ, ground blank samples (10 replicates) were spiked at 1 µg kg^−1^ using the appropriate volume of the working standard solution. The 1 µg kg^−1^ spiked concentration was based on the maximum level of atropine and scopolamine permitted in baby foods and processed cereal-based food for infants and young children allowed by the Commission Regulation (EU 2023). The LOD and LOQ were estimated using the standard deviation (SD) of replicate measurements (*n*) following the Eurachem guide (Eqs. 3 and 4) (Magnusson 2014):

3 $${\text{LOD}} = { }\left( {{\text{SD}}\sqrt n } \right) \times 3$$

4 $${\text{LOQ}} = { }\left( {{\text{SD}}\sqrt n } \right) \times 10$$

### TA Transfer Study

Soybeans were purchased from a local supermarket and tested for TAs (atropine and scopolamine) using the optimised and validated SLE-DnS-LC-MS/MS method. Soybeans with atropine and scopolamine content < LOD were designated as blank soybeans and subsequently used for TA transfer study. Fourteen (10 g each) soybean samples were placed in individual plastic containers with a lid. Each soybean sample was contaminated with 0.1% dried seeds of *D. stramonium* and incubated for a total of 0, 15, 90, 180, 360, 1440 or 2880 minutes (in duplicate) with shaking at 2500 rpm. Following each incubation period, *D. stramonium* seeds were removed and soybeans were ground and mixed thoroughly, and a 1 g portion was subjected to extraction of TAs following the procedure in Section “Sample Extraction Procedure”.

### TA Exposure Study

Based on the contents of TAs (atropine plus scopolamine) in soybeans determined in this study, the estimated daily intake (EDI) of TAs was calculated using the per capita consumption of soybeans (Eq. 5) (EFSA and Binaglia 2022). In addition, the margin of exposure (MOE) to assess the health risk of TA exposure in soybeans was also calculated (Eq. 6), considering a BMDL_05_ of 1.54 µg kg^−1^ body weight per day (EFSA et al. 2022).

5 $${\text{EDI}}\frac{{ \mu {\text{g}}}}{{{\text{day}}}} = \frac{{{\text{g}} \,{\text{soybean}}}}{{{\text{day}}}} \times \frac{{1 \,{\text{kg}}}}{{1000 {\text{g}}}} \times \frac{{\mu {\text{g}} \,{\text{atropine}}\, + \,{\text{scopolamine}}}}{{{\text{kg}} \,{\text{soybean}}}}$$

6 $${\text{MOE}} = { }\frac{{{\text{Benchmark}}\,{\text{ dose }}\,{\text{level }}\,\left( {{\text{BMDL}}} \right)}}{{{\text{Estimated }}\,{\text{Daily}}\,{\text{ Intake}}\,{ }\left( {{\text{EDI}}} \right)}}$$

## Results and Discussion

### LC-MS/MS Optimisation

Three columns were tested during the LC method optimisation to provide optimal resolution of both compounds: Phenomenex Gemini C18 reversed-phase column (100 × 4.6 mm, 5 μm particle size, 110 Å), Kinetex F5 column and Luna Omega polar C18 column with the exact specifications (100 × 4.6 mm, 5 μm particle size, 110 Å). Among these three tested columns, the Phenomenex Gemini C18 resulted in a satisfactory separation of atropine and scopolamine, showing well-resolved peaks of the two TAs, which proved better than the other two columns.

Two mobile phases were compared: (1) methanol: water: acetic acid (10:89:1, v/v/v) (MPA) and methanol: water: acetic acid (97:2:1, v/v/v) (MPB) both containing 5 mM ammonium acetate buffer *vs* (2) water with 0.1% formic acid (MPA) and methanol with 0.1% formic acid (MPB). The initial mobile phases (1) tested were routinely used in the authors’ research laboratory for analysing plant toxins and mycotoxins. Therefore, a comparison was made between two different mobile phases (1 *vs* 2). Consistent with other published methodologies (Begemann et al. 2021; Haughey et al. 2021), the latter mobile phases, specifically H_2_O with 0.1% formic acid (MPA) and methanol with 0.1% formic acid (MPB), were selected. These mobile phases provided better separation between atropine and scopolamine, enhanced the signal-to-noise (S/N) ratio for each analyte, and were easier to prepare (see Supplementary Figure 1).

### Optimisation of the Extraction Procedure

The extraction technique employed in this study was an ultrasound-assisted SLE with DnS as the only clean-up step, a slightly modified method for mycotoxins analysis based on Meneely et al. (2023). In the DnS technique, the sample extract is diluted with a suitable solvent, typically with the same composition as the extraction solvent but with different proportions, followed by mixing (in a vortex) to facilitate solubilisation and homogenisation of analytes into the solvent, centrifugation, and filtration, before injection into the LC-MS system (Greer et al. 2021). Although some extraction methods that involve clean-up steps, like SPE and QuECHERS can enhance the method's sensitivity (Marín-Sáez et al. 2017; Vuković et al. 2022), they may be less practical for routine analysis involving hundreds or even thousands of samples, as in the current study. Commercial cartridges and salts can be costly, making a straightforward and less expensive approach, such as DnS, more desirable. In DnS, the extraction steps are simplified due to the absence of pre-concentration, evaporation and reconstitution steps, which are often time-consuming and tedious. Lastly, DnS enables high throughput without requiring specialised equipment.

TAs are soluble in aqueous and acidified organic solvents (Begemann et al. 2021; Chen et al. 2017; Vuković et al. 2022). Acetonitrile or methanol is the most commonly used organic solvent, and formic or acetic acid is generally used as the additive for acidification (Begemann et al. 2021; Chen et al. 2017; Vuković et al. 2022); consequently, various combinations were investigated in this study to achieve the optimal extraction solvent. Preliminary evaluation of the extraction solvent was based on the extraction recovery (%) and the response of atropine and scopolamine (peak intensity) to a spike concentration level of 1 µg kg^−1^ in ground homogenised blank soybean sample (five replicates for each extraction solvent tested). A 1 µg kg^−1^ concentration was chosen based on the lowest maximum level legally permitted in cereal-based foods for infants and young children (EU 2023).

The four extraction solvents investigated in the optimisation included an in-house solvent composed of acetonitrile: water: acetic acid (79:20:1, v/v/v), which was previously used for the extraction of mycotoxins by Meneely et al. (2023), and three extraction solvents with varying concentrations of acetonitrile (60, 70 and 80%) and 1% formic acid (v/v). These solvents were compared to determine the most effective extraction solvent for the TA analysis.

The extraction involved 90 min of shaking before centrifugation (for 15 min at 2516 RCF (g force)), dilution of the supernatant, and filtration before injection into the LC system. Among the four extraction solvents tested, the acetonitrile: water (60:40, *v/v*) with 1% formic acid gave the best efficiency of extraction, which was close to the acceptable lower limit of 70% (EURL 2021). The composition of the optimum extraction solvent was similar to what others used in the study of TAs in foods (Gonçalves et al. 2020; Haughey et al. 2021; Veršilovskis et al. 2020).

Ultrasonication (for 30 min) was also performed following the 90-min shaking on the multi-tube vortex to improve the extraction recovery. With sonication added to the extraction procedure, the extraction recovery improved for both analytes, being higher than 85% for atropine and 81% for scopolamine in soybean (Table 3). Although the extraction time is 140 min, it allows for the simultaneous extraction of at least 60 samples, effectively reducing the extraction time to just 2.3 min per sample, making it both efficient and scalable. Ultrasound-assisted extraction (sonication or ultrasonication) involves acoustic cavitation in a liquid medium due to the propagation of soundwaves, leading to the disruption and erosion of the plant cell walls and membranes (Kumar et al. 2021; Ranjha et al. 2021) facilitating the release of intracellular contents or improving the penetration of the solvent into the plant matrix and enhancing the mass transfer of the extracted compounds from the solid phase to the liquid phase (Kumar et al. 2021; Ranjha et al. 2021). Extraction time plays a crucial role in determining the yield of TAs during solvent extraction. For instance, TA extraction recovery from *D. innoxia* using methanol increased up to four hours, but no significant increase was observed longer than four hours (Yohannes et al. 2019). The optimal extraction time depends on various factors such as the extraction technique, solvent system, plant material, and target analytes (Yohannes et al. 2019).

**Table 3:** Analytical performance of the ultrasound-assisted solid-liquid extraction with dilute-and-shoot coupled to LC-MS/MS method for the quantification of atropine and scopolamine in soybean matrix (determination in five replicates)

Parameter	Atropine	Scopolamine
LOD (µg kg^−1^)	0.03	0.08
LOQ (µg kg^−1^)	0.10	0.25
*Intra-day precision (%RSD*_*intra*_*)*		
1 µg kg^−1^	3	6
5 µg kg^−1^	7	8
50 µg kg^−1^	1	2
*Inter-day precision (%RSD*_*inter*_*)*		
1 µg kg^−1^	11	11
5 µg kg^−1^	12	10
50 µg kg^−1^	8	7
*Extraction recovery (%)*		
1 µg kg^−1^	98	99
5 µg kg^−1^	93	91
50 µg kg^−1^	85	81
Linearity range* (*R*^2^)	0.9992	0.9989
Slope of matrix calibration (m)	64765	50425
Slope of solvent calibration (m)	62653	47266
Matrix effect (SSE) (%)	3.4	6.7

*Linearity range: Atropine= 0.10–100 µg kg^−1^; scopolamine= 0.25–100 µg kg^−1^

### Method Validation

A matrix-matched calibration curve was prepared by adding appropriate standard solutions containing atropine and scopolamine in blank sample extracts, obtaining final concentrations of 0.10, 0.25, 0.5, 1.0, 2.5, 5, 10, 25, 50, and 100 µg kg^−1^. Although preparing matrix-matched calibration curves can be time- and resource-intensive, particularly when analysing various matrices, the simplicity of the DnS method facilitates easier execution of matrix-matched calibration preparation. The calibration curves for atropine and scopolamine had a coefficient of determination (*R*^2^) ≥ 0.99 for all matrices studied (soybean: Table 3; corn, millet, sorghum, wheat, Super Cereals, and Super Cereal Plus: Table 4). The *R*^2^ shows that the developed method for quantifying atropine and scopolamine had a strong linear relationship between the different concentrations in the matrix-matched calibration and the signal intensity (area). Regulatory guidelines such as the International Council for Harmonisation of Technical Requirements for Pharmaceuticals for Human Use (ICH) (FDA 2022) recommend an *R*^2^ ≥ 0.99 as evidence of acceptable linearity for most analytical methods.

**Table 4:** Extraction recovery and matrix effect on atropine and scopolamine of corn, millet, sorghum, wheat, Super Cereals, and Super Cereal Plus

Matrix	Intra-day precision (%RSD)			Extraction recovery (%)			Linearity range* (*R*^2^)	Matrix slope	Solvent slope	Matrix effect (%)
	1 µg kg^−1^	5 µg kg^−1^	50 µg kg^−1^	1 µg kg^−1^	5 µg kg^−1^	50 µg kg^−1^				
*Corn*										
Atropine	2	4	2	112	108	106	0.9998	108,009	102,033	6
Scopolamine	3	5	2	92	104	106	0.9998	71,004	76,693	7
*Millet*										
Atropine	5	3	3	99	108	101	0.9999	81,075	74,316	9
Scopolamine	4	3	6	108	118	108	0.9999	44,012	60,039	27
*Sorghum*										
Atropine	9	10	1	110	108	87	0.9998	96,716	104,899	8
Scopolamine	10	5	2	109	112	96	1.000	54,994	77,967	30
*Wheat*										
Atropine	9	2	2	117	97	99	0.9999	99,653	103,912	4
Scopolamine	5	2	3	106	93	102	0.9999	57,904	70,059	17
*Super Cereals*										
Atropine	6	2	1	93	85	86	0.9999	99,967	103,443	3
Scopolamine	5	2	2	85	83	84	0.9995	61,387	74,968	18
*Super Cereals Plus*										
Atropine	8	10	10	95	101	106	0.9999	77,895	82,334	5
Scopolamine	8	9	15	94	107	107	0.9999	53,588	65,176	18

*Linearity range: Atropine= 0.10–100 µg kg^−1^; scopolamine= 0.25–100 µg kg^−1^

Matrix effects were calculated as outlined above by comparing the slope of the regression curve obtained for the chosen analytes in solvent only *vs* the slope of the regression curve for the selected analytes in the matrix (Eq. 2). Matrix effects are described as the decrease or increase of the analytical response of a given analyte due to co-eluting matrix constituents, which is inherent to LC-MS-based methods for the analysis of dilute crude extracts (Sulyok et al. 2020). The matrix effects were ≤ 20% for soybean, wheat, corn, Super Cereals or Super Cereal Plus, indicating the absence of a significant matrix effect (Tables 3 and 4). Hence, a solvent (external) calibration curve could accurately quantify TAs in these matrices as the extraction recovery was high and the SSE was low. The sorghum and millet samples demonstrated an acceptable matrix effect on atropine of less than 20%. The matrix effect on scopolamine was 27% and 30% for each sample, respectively. This is considered insignificant, especially given the high extraction recoveries, suggesting that the losses were due to the SSE alone, in addition to an RSD of less than 10%, indicating the robustness of the current method (Table 4). Matrix effects can vary between different compounds due to their distinct chemical properties and interactions with the matrix components (González-Gómez et al. 2022; Sulyok et al. 2020), which explains the differences between atropine and scopolamine in sorghum or millet. Furthermore, there is no clear-cut guideline on an acceptable range of matrix effects for food analysis (Sulyok et al. 2020). Based on the matrix effect classification measured by the response of the matrix-matched standard compared with the solvent standard (Sulyok et al. 2020), all matrices had soft suppression (matrix effect of ± 20%), except for sorghum and millet, which had a moderate suppression effect (± 50% to ± 20%) on scopolamine (Tables 3 and 4).

Performance data at three spike concentration levels of atropine and scopolamine (1, 5, and 50 µg kg^−1^) on soybean matrix had recoveries of 85–98% for atropine and of 81–99% for scopolamine, with RSD_intra_ and RSD_inter_ of ≤ 12% (Table 3), fulfilling the recommended limits of 70–120% set by SANTE/11312/2021 (EURL 2021). Similarly, the extraction recoveries of atropine and scopolamine in corn, sorghum, wheat, Super Cereals, and Super Cereal Plus were between the limits set by SANTE/11312/2021 and with RSD_intra_ ≤ 15% (Table 4). The RSD% values corresponding to intra- and inter-day precision below ≤ 20% were considered acceptable as per SANTE/11312/2021 (EURL 2021).

The LODs for atropine and scopolamine were 0.03 and 0.08 µg kg^−1^, respectively, and the LOQs were 0.10 and 0.25 µg kg^−1^, respectively (Table 3). The LOQ is lower than the maximum limit of 1 µg kg^−1^ for atropine or scopolamine set by Commission Regulation (EU) 2023/915 (EU 2023) for food and processed cereal-based food for infants and young children containing millet, sorghum, buckwheat, corn or their derived products, or 5 µg kg^−1^ for unprocessed millet grains and sorghum grains, and corn for popping, millet, sorghum and corn placed on the market for the final consumer, as well as for milling products of millet, sorghum and corn. The sensitivity of the current method was comparable to that of Marín-Sáez et al. (2017) (LOQ-1–2 µg kg^−1^) that employed a strong cationic exchange sorbent for SPE for cereal samples, and González-Gómez et al. (2020) (LOQ= 1.5 and 2.4 µg kg^−1^ for atropine and scopolamine) for gluten-free grains or flours, or even with QuEChERS for atropine and scopolamine (LOQ= 0.5 µg kg^−1^) in cereal samples (Baslé et al. 2020). However, the LOQ (0.1–0.25 µg kg^−1^) attained in the current study was higher than those from Begemann et al. (2021) (LOQ= 0.01 and 0.025 ng mL^−1^). Despite the current research not using SPE cartridges to clean or concentrate the extracts, and with only the DnS as a clean-up procedure following SLE, the LOD and LOQ are suitable for detecting and quantifying the levels of atropine and scopolamine in the matrices analysed (cereals, oilseeds and products), as required by the EU, demonstrating that the method is fit-for-purpose.

### Application of the Method

Implementing reliable and accurate methods to detect and quantify TAs in crops and food products is essential for monitoring the presence of these toxic compounds and establishing strategies to mitigate or reduce the risk of poisoning humans and animals following their consumption. The DnS-LC-MS/MS method optimised and validated in this study was applied to the determination of atropine and scopolamine in 331 soy (269 seeds and 62 meal) samples collected from 13 countries between 2021 and 2024. There were also 42 Super Cereals from various suppliers, 39 of which were involved in the Uganda poisoning incident in 2019 (Haughey et al. 2021), and 40 uncooked and 40 cooked millet or corn from Cameroon were analysed in this study.

**Fig. 1:**
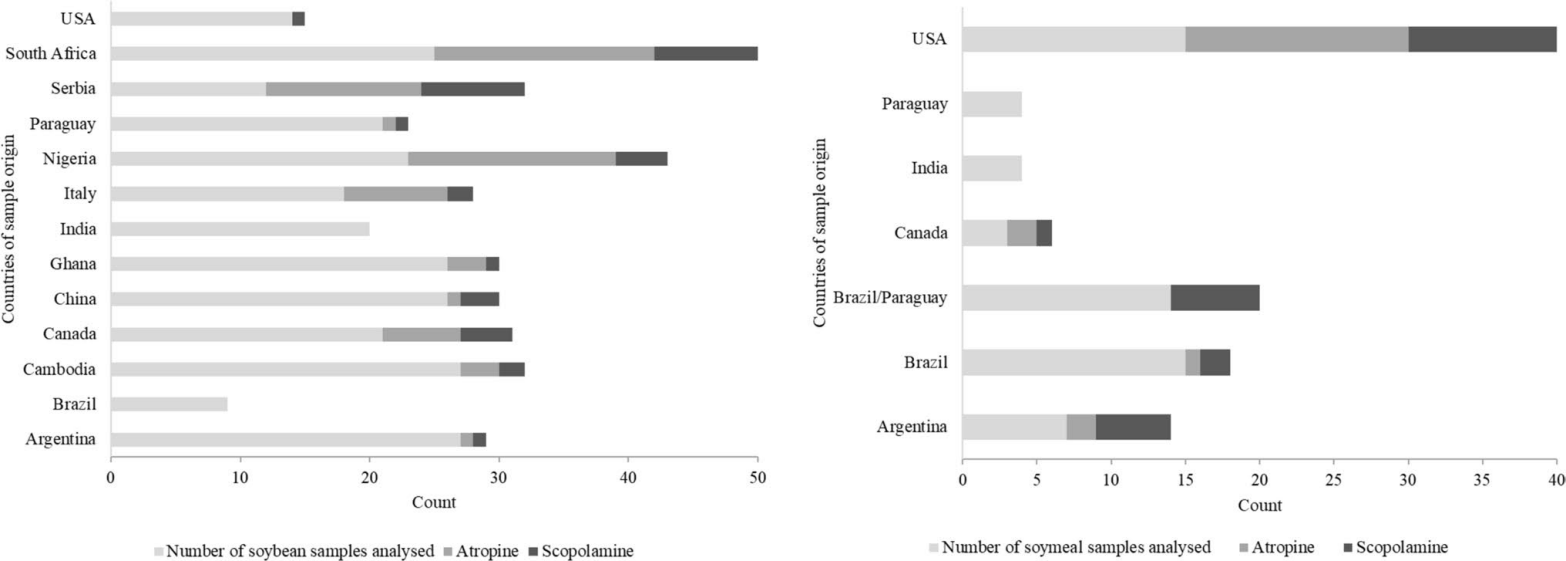
Soybean (**left)** and soymeal (**right)** samples that have concentrations of atropine and scopolamine at and above the limit of quantification (LOQ). (LOQ_atropine_= 0.10 μg kg^−1^; LOQ_scopolamine_= 0.25 μg kg^−1^)

Of the 269 soybean samples analysed, atropine was present (≥ LOQ of 0.10 µg kg^−1^) in 69 samples, and scopolamine was present (≥ LOQ of 0.25 µg kg^−1^) in 35 samples (Fig. 1; Supplementary Table 2). Atropine was present (≥ LOQ) in all soybean samples from Serbia (12 of 12), 70% of samples from Nigeria, and 68% of samples from South Africa. On the other hand, 67%, 32% and 19% of soybean samples from Serbia, South Africa and Canada, respectively, contained scopolamine (≥ LOQ). Neither atropine nor scopolamine was detected in soybean samples from Brazil and India (LOD atropine and scopolamine were 0.03 and 0.08 µg kg^−1^, respectively).

**Fig. 2:**
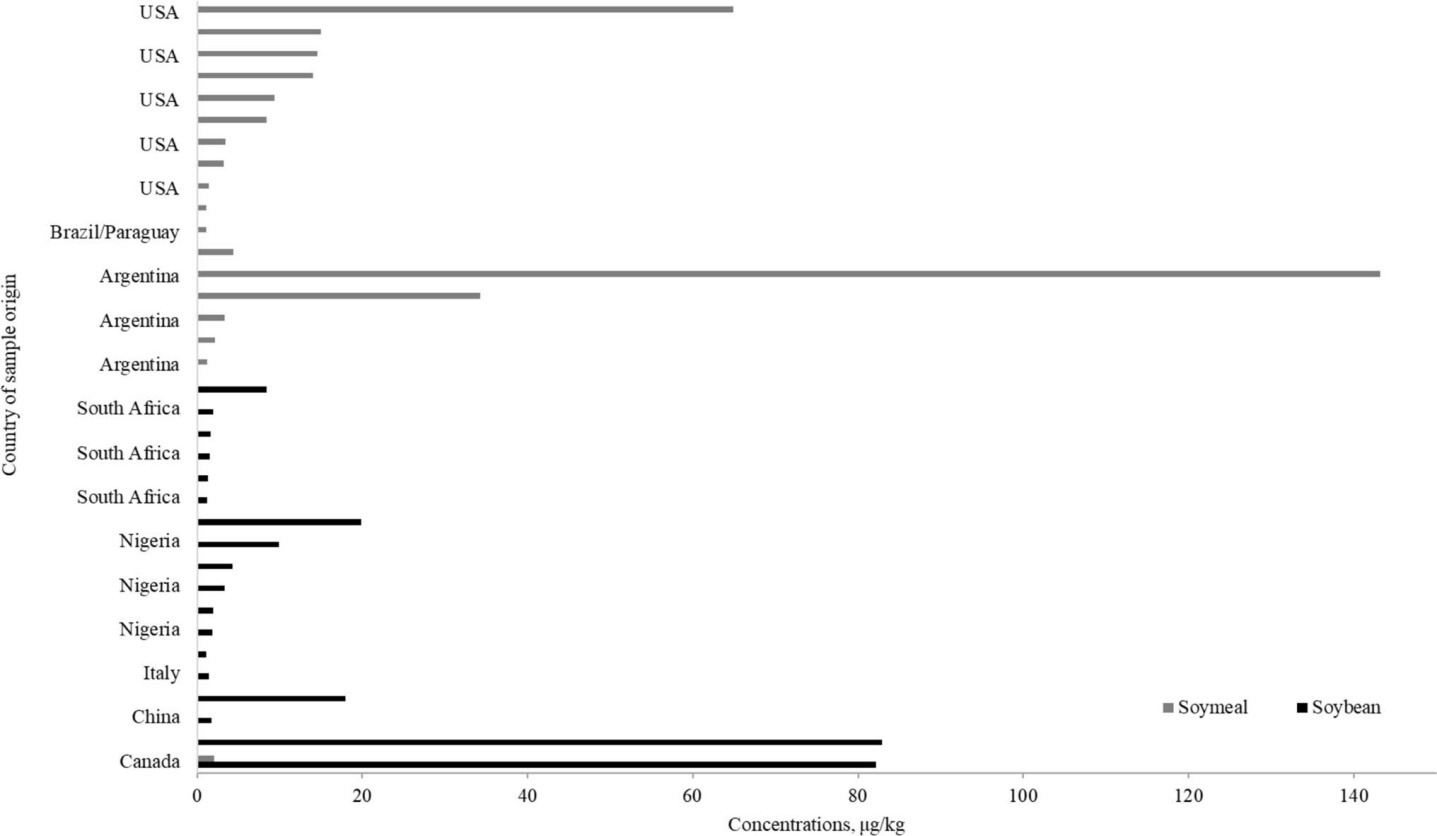
The sum of atropine and scopolamine in soybean or soymeal samples exceeded the maximum limit of 1 μg kg^−1^ set by the EU (an additional soymeal sample from the USA contained 388.7 μg kg^−1^)

High levels of TAs in foods have resulted in poisoning cases and intoxication, by which atropine and scopolamine, as dose-dependent anticholinergics or as competitive antagonists of the muscarinic acetylcholine receptors, with symptoms including decreased secretion from salivary glands, dilation of the pupils and increased heart rate (EFSA et al. 2022). At toxic levels, they stimulate the central nervous systems with restlessness, disorientation, hallucinations and delirium. At the same time, at much higher doses, there is central depression leading to death from respiratory paralysis (EFSA et al. 2022; FAO/WHO 2020). The EFSA Panel on Contaminants in the Food Chain (CONTAM Panel) published a scientific opinion on the risks to human and animal health related to the presence of TAs in food and feed, and a group acute reference dose (ARfD) of 0.016 μg kg^−1^ body weight for the sum of atropine and scopolamine, assuming equivalent potency (EFSA et al. 2022). Currently, there is no regulation for the levels of atropine and scopolamine in oilseeds (e.g., soy), but assuming that the maximum level for the sum of atropine and scopolamine in soybeans is 1 µg kg^−1^, there were 18 of 269 (6.7%) soybean samples at or above this permitted value from five countries (Canada, China, Italy, Nigeria and South Africa) (Fig. 2). The sum of atropine and scopolamine was the highest in the two Canadian samples, with 82.15 and 82.93 µg kg^−1^ (Fig. 2; Supplementary Table 3). The estimated daily intake (EDI) of atropine and scopolamine, along with the margin of exposure (MOE), were calculated using Eqs. (5) and (6), respectively (EFSA et al. 2022). These calculations considered the estimated per capita soybean consumption of 15.3 kg per year for Canada, 9.37 kg for China, 4.27 kg for Italy (based on EU per capita consumption), 0.60 kg for Nigeria, and 0.04 kg for South Africa. MOE values less than 100 indicate a potential public health concern associated with the presence of a toxic compound in the cereal-based foods, while MOE values above 100 suggest that there is no significant public health concern regarding the toxic compound in the cereals (EFSA 2023). Of the 18 samples with atropine and scopolamine above the maximum limit, seven samples (two from Canada, China or Nigeria, and one from Italy) had MOE of less than 100, and therefore, there is a potential public health concern.

There were 20 of 62 soymeal samples that had atropine (≥ LOQ of 0.10 µg kg^−1^) and 24 of 62 soymeal samples that had scopolamine (≥ LOQ of 0.25 µg kg^−1^); the highest content being a soymeal sample from the USA (274.52 µg kg^−1^ atropine) and Argentina (141.38 µg kg^−1^ scopolamine). Neither atropine nor scopolamine was detected in soymeal samples from Brazil/Paraguay, Paraguay, and India (Fig. 1, Supplementary Table 2). Regarding soymeal, 19 of 62 samples (30%) had the sum of atropine and scopolamine above the assumed maximum level of 1 µg kg^−1^ (Fig. 2, Supplementary Table 3). These soymeal samples came from Argentina, Brazil, Paraguay, Canada and the USA. The highest concentration for the sum of atropine and scopolamine was found in soymeal from the USA (388.77 µg kg^−1^), followed by a soymeal sample from Argentina (143.19 µg kg^−1^) (Fig. 2, Supplementary Table 3). Although soymeal is primarily for animal consumption, the general presence of TAs in soybeans (6.7%) at levels beyond the maximum limit of 1 µg kg^−1^ requires increased monitoring to better understand human exposure to TAs.

List (1976) added finely ground *D. stramonium* seeds to dehulled and flaked soybeans; 90% of the atropine and 96% of the scopolamine partitioned into the defatted soybean fraction during oil extraction. This demonstrates that if *D. stramonium* contamination occurs, the TAs preferentially concentrate in the defatted soybean meal (or soymeal) rather than the oil fraction during processing. Soybean meal is rich in protein and essential amino acids. It is vital in both the animal feed industry and the human food industry. In the food industry, soymeal helps manufacture soy protein concentrates/isolates and texturised vegetable proteins, which are used as ingredients for meat alternatives; hence, monitoring their quality and safety before use is crucial.

Of the 40 cooked and 40 uncooked corn or millet samples from Cameroon, atropine or scopolamine was detected in nine samples (11%), but only one sample contained TAs above the maximum level of 1 µg kg^−1^. Malysheva et al. (2023) reported that the contamination of the buckwheat, sorghum, and millet products was low to non-existent. On the other hand, TA contamination has been frequently associated with cereal and bakery products, as reported by the Rapid Alert System for Food and Feed (RASFF) (FOODAKAI 2024). Despite heat treatment, broa de milho, a traditional bread made from corn, has caused hundreds of people to become ill in Portugal, and Super Cereals, a processed cereal product prepared with heat treatment, has caused poisoning and death of many people in Uganda (Gravador et al. 2024). The effects of cooking on TAs are inconclusive, with some studies showing reductions in TA contents at high temperatures, or interconversion or transformation into other products, such as low molecular weight TAs or other degradation products, which are even more toxic (Casado et al. 2023). Hence, it was advised to have more in-depth studies to better explain the impacts of cooking or heat treatment of TAs, and the degradation products, and perhaps some regulation is needed for these compounds (Casado et al. 2023).

Three of the 42 Super Cereals samples did not contain scopolamine (<LOD of 0.08 µg kg^−1^). Thirty-nine of these Super Cereals were involved in the food poisoning incident in Uganda in 2019, where about 300 people were affected (Abia et al. 2021; Haughey et al. 2021). Super Cereal and Super Cereal Plus are used in humanitarian programmes/response; they are fortified flour of corn or wheat and soy—prepared from heat-treated corn and de-hulled soya beans, sugar, dried skim milk, and refined soya bean oil (FAO/WHO 2020). The recommended levels for the sum of atropine and scopolamine were 30 μg kg^−1^ for children (from 5 years of age) and adults for Super Cereals, and 10 μg kg^−1^ for infants and young children (6–59 months old) for Super Cereal Plus. However, in situations such as food insecurity, the guidance levels are increased to 90 μg kg^−1^ for Super Cereals and 30 μg kg^−1^ for Super Cereal Plus (FAO/WHO 2020). Twenty Super Cereals had atropine and scopolamine levels greater than or equal to the recommended 30 µg kg^−1^. In these 20 Super Cereals, the mean atropine and scopolamine content was 14,168 µg kg^−1^, with the highest and lowest concentrations being 53,051 µg kg^−1^ and 32 µg kg^−1^, respectively. In an earlier review on TA poisoning incidents (Adamse et al. 2014), it was cited that the estimated lethal dose of atropine in humans is 10 mg and 2–4 mg of scopolamine. Consequently, consumption of these products caused hundreds of people in Uganda to become ill and resulted in five deaths (Abia et al. 2021; Haughey et al. 2021).

The food factory that produced the Super Cereals, which was involved in the Uganda TA food poisoning incident, held ISO 9001 and ISO 22000 certifications in 2018. However, an inspection conducted by CWA International Ltd. revealed significant deficiencies in Good Agricultural Practices (GAP), Good Manufacturing Practices (GMP), and quality management systems, such as Hazard Analysis and Critical Control Points (HACCP) (Abia et al. 2021). For example, the raw materials used did not meet the specifications, such as the soybeans, which were of animal feed grade (Abia et al. 2021; Haughey et al. 2021). The EU Directive 2002/32/EC states that animal feed is allowed to contain weed seeds and unground and uncrushed fruits containing alkaloids, glucosides, or other toxic substances separately or in combination with a maximum level of 3,000 mg kg^−1^, including *Datura* sp. with a maximum level of 1000 mg kg^−1^ (Abia et al. 2021; EC 2002). These levels are very high for human consumption, specifically that the recommended TA levels for Super Cereals are only between 30 and 90 μg kg^−1^. Further to the observation of CWA International Ltd., there were no records of grain cleaning, and the process lacked adequate sieving to ensure the separation of TA plant sources from the food grains. Additionally, the factory and equipment were not routinely cleaned, there was no evidence of TA analysis on raw materials or final products, and staff training and education were insufficient (Abia et al. 2021).

### Transfer of TAs in Soybeans

**Fig. 3:**
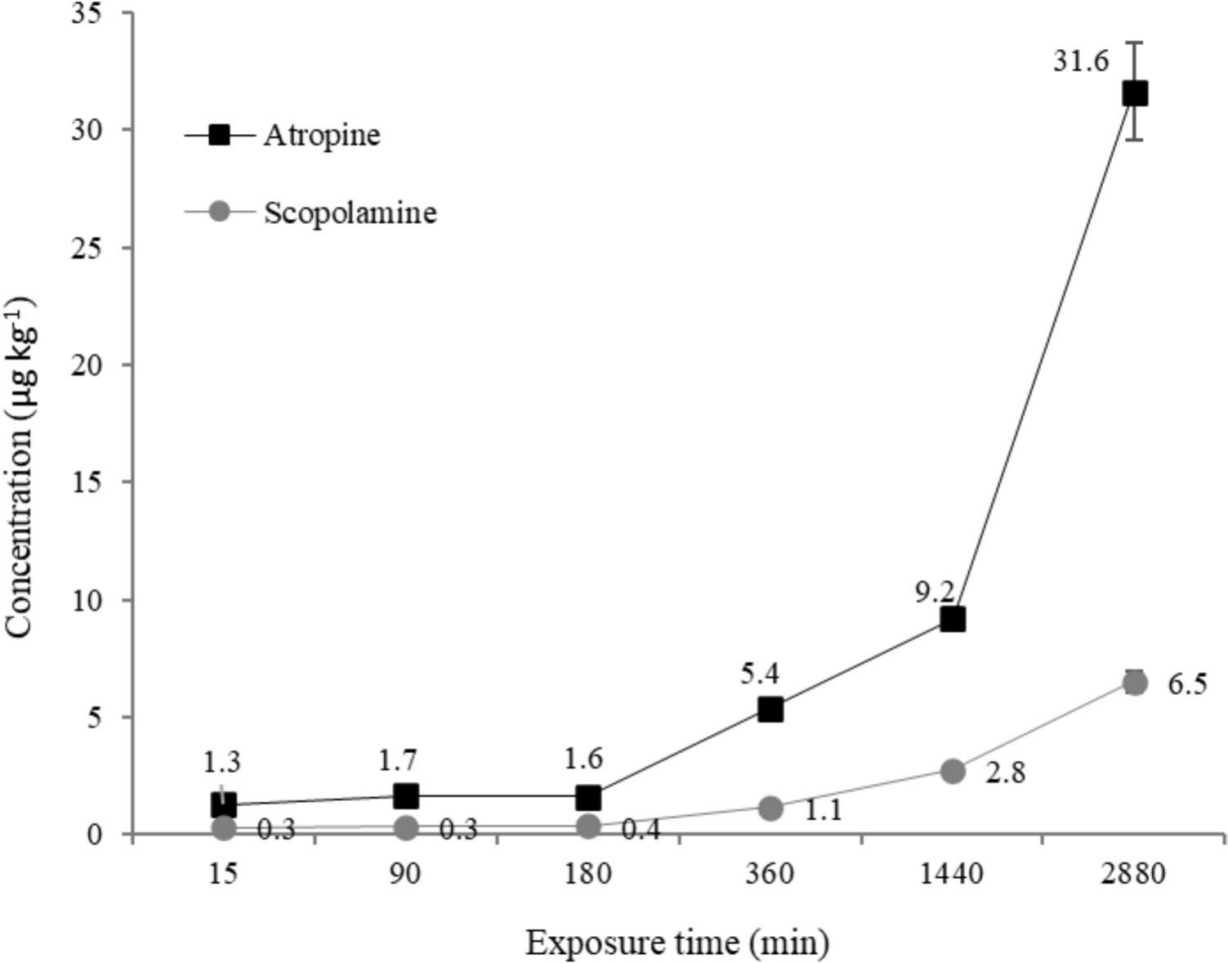
Changes in the concentrations of atropine and scopolamine (μg kg^−1^) in soybeans exposed to *Datura stramonium* seeds (0.1%) at different times

During visual inspection, the 269 soybean samples collected from 13 different countries were received as clean, whole soybean seeds, with no contaminating foreign plant materials present; thus, we hypothesised that the contamination may have come from the invisible transfer of atropine and scopolamine from the TA plant sources to soybeans. To confirm the possible transfer of atropine and scopolamine through contact of soybeans with the TA plant source, blank soybean samples (with no detected atropine and scopolamine or < LODs) were exposed to *D. stramonium* seeds (0.1%) with agitation at different incubation periods. After each incubation period, *D. stramonium* seeds were removed, the soybeans were ground, and the contents of atropine and scopolamine were determined using the LC-MS/MS method optimised in this study. The results (Fig. 3) showed that after 15 min of exposure (shaking at 2500 rpm) to *D. stramonium* seeds, atropine concentration reached 1.3 µg kg^−1^ and scopolamine at 0.3 µg kg^−1^. After 360 min (6 h), the atropine and scopolamine levels were 5.4 and 1.1 µg kg^−1^, respectively. This could explain the presence of atropine and scopolamine in soybean samples despite the absence of *Datura* seeds in the soybean, in agreement with a previous study by Begemann et al. (2021).

As the EU RASFF indicates, *Datura* species are commonly found as contaminants in food crops. These invasive plant species thrive in many temperate regions of Asia and have spread to the United States, Canada, and the West Indies (Abia et al. 2021; de Nijs et al. 2023). The spread and distribution of these weeds are influenced by environmental factors, particularly extreme weather and rising temperatures due to climate change (Abia et al. 2021; de Nijs et al. 2023). *D. stramonium* is increasingly prevalent in crops such as corn, millet, sorghum, and soybeans, entering the food chain through unintentional mixing during harvesting and processing.

The entire *D. stramonium* plant contains TAs, i.e., from its roots, stem, leaves, flowers, fruits and seeds, hence mixing with any of these can result in TA contamination. *Datura* plants germinate later, and crop contact with their sap during harvest, especially mechanically (Abia et al. 2021). While *D. stramonium* seeds can be mechanically screened, sorted and removed, separation becomes challenging when crop seeds are of similar size to *Datura* seeds (e.g., buckwheat). Furthermore, despite the TA plant removal or cleaning of food grains following harvest, TA can remain in dust or invisibly transferred through abrasion to the crop; therefore, sorting or cleaning may not always prevent TA presence in food crops (Begemann et al. 2021). Consequently, in this situation, it is critical to prevent the mixing of TA plant sources with the harvested crops; thus, GAP, GMP and food quality management systems are critical, as emphasised by the World Health Organisation, and consequent to the Uganda poisoning incidents (Abia et al. 2021; de Nijs et al. 2023). Application of herbicides to prevent the growth of *Datura* plants in the agricultural field could be a straightforward strategy. However, the strict legislation governing their application to control the residue limits in food crops hinders their use by producers (Abia et al. 2021; Kudsk 2020); nonetheless, understanding the regulations and authorised pesticides to use in the field could be helpful to tackle invasive TA-producing plants. Additionally, educating/training farmers to identify, separate, routinely check and dispose of these toxic plants from the fields and at harvest, and sorting them at harvest but before processing, will certainly reduce TA contamination of food crops (Abia et al. 2021; Adamse et al. 2014; Alletsee 2016).

## Conclusion

The current study aimed to optimise and validate ultrasound-assisted SLE with DnS as a clean-up step for quantifying scopolamine and atropine in soy (soybean and soymeal), cereals (corn, wheat, millet and sorghum) and products (Super Cereals and Super Cereal Plus) using LC-MS/MS. The method meets all the criteria outlined in international validation guidelines, making it suitable for monitoring the levels of atropine and scopolamine in food grains (cereals, oilseeds, and their products) to ensure the safety of food along the supply chain. The method demonstrated excellent percentage recoveries of atropine and scopolamine and minimised matrix effects. Thus, it showed insignificant signal enhancement or suppression and a linear response from 0.10–100 µg kg^−1^ in either matrix-matched or external calibration curves. The LOQs were 0.10 µg kg^−1^ for atropine and 0.25 µg kg^−1^ for scopolamine, which were lower than the maximum level permitted in cereals and related products set by the EU Commission Regulation 2023/915 (1 µg kg^−1^ or 5 µg kg^−1^, depending on the commodity), showing that the method is fit for purpose. The SLE procedure with DnS is cost-effective and straightforward since no sophisticated equipment, costly clean-up steps, or specialised skills are required. The method is high-throughput since at least 60 samples can be extracted within 140 min, equivalent to 2.3 min per sample, with each sample requiring only 8.5 min for LC-MS/MS analysis. Of the 39 Super Cereals involved in the Uganda poisoning incidents, 20 had atropine and scopolamine content beyond the recommended levels. The analyses of an extensive collection of soybeans/soymeal from 13 different locations globally showed that 6.7% of soybean samples, and 31.7% of soymeal samples had the sum of atropine and scopolamine above the permitted level (1 µg kg^−1^) . Based on the per capita consumption of soybeans, 2.6% had an MOE below the acceptable limit, indicating higher risks and potential health concerns. Contamination of food commodities by TAs does not need the presence of TA-producing plant (e.g., seeds of *D. stramonium*). The results of this study have shown that exposing food grains to *D. stramonium* seeds for an extended period can transfer atropine and scopolamine, resulting in levels beyond the regulated limit (1 µg kg^−1^). Based on the nature and toxicity of TA-producing plants, it is best to prevent them from entering the food supply chain from the field through GAP. Research is needed to determine cleaning procedures that can reduce TA levels in contaminated harvested crops, especially when mixing cannot be avoided in the field. Nevertheless, based on the results, monitoring strategies must be developed, and GAP, GMP and Food Safety Management Systems must be in place to protect consumers’ safety against these harmful atropine and scopolamine.

## Supplementary Information

Below is the link to the electronic supplementary material.

Supplementary file1 (DOCX 141 kb)

## Data Availability

Data are available in this paper and/or from the corresponding author upon reasonable request.
