# Supplementary material for: Determination of Toxic Tropane Alkaloids in Globally Sourced Soya, Cereals and Products Using Dilute-and-Shoot Technique Coupled with LC-MS/MS
Source: Expo Health. 2025 Jul 1;17(5):1193–206. doi: 10.1007/s12403-025-00718-z (PMC12436479; doi:10.1007/s12403-025-00718-z)

### Supplementary Table 1. Country of origin of the soy samples used in this study.

| Country of origin | Soybean | Soymeal |
| --- | --- | --- |
| Argentina | 27 | 7 |
| Brazil | 9 | 15 |
| Brazil/Paraguay | 0 | 14 |
| Cambodia | 27 | 0 |
| Canada | 21 | 3 |
| China | 26 | 0 |
| Ghana | 26 | 0 |
| India | 20 | 4 |
| Italy | 18 | 0 |
| Nigeria | 23 | 0 |
| Paraguay | 21 | 4 |
| Serbia | 12 | 0 |
| South Africa | 25 | 0 |
| USA | 14 | 15 |

Supplementary Table 2. The concentrations (µg kg^-1^) of atropine and scopolamine in soy samples analysed from 13 different regions globally.

| Country of origin | Soybean | | | | |  | Soymeal | | | | | |
| --- | --- | --- | --- | --- | --- | --- | --- | --- | --- | --- | --- | --- |
|  | Number of samples analysed | Atropine | | Scopolamine | |  | Number of samples  analysed | Atropine | | Scopolamine | | |
|  |  | Number of samples ≥ LOQ (0.10 µg kg^-1^) | concentration range  (µg kg^-1^) | Number of samples ≥ LOQ (0.25 µg kg^-1^) | concentration range  (µg kg^-1^) |  |  | Number of samples ≥ LOQ (0.10 µg kg^-1^) | concentration range  (µg kg^-1^) | Number of samples ≥ LOQ (0.25 µg kg^-1^) | | concentration range  (µg kg^-1^) |
| Argentina | 27 | 1 | 0.18 | 1 | 0.02-0.25 |  | 7 | 2 | 0.75-1.81 | 5 | | 1.24-141.38 |
| Brazil | 9 | 0 |  | 0 | 0.08-0.13 |  | 15 | 1 | 0.2 | 2 | | 0.08-4.35 |
| Brazil/Paraguay | 0 |  |  |  |  |  | 14 | 0 |  | 6 | | 0.09-1.10 |
| Cambodia | 27 | 3 | 0.18-0.57 | 2 | 0.16-0.30 |  | 0 |  |  |  | |  |
| Canada | 21 | 6 | 0.20-70.11 | 4 | 0.12-18.83 |  | 3 | 2 | 0.24-1.62 | 1 | | 0.41 |
| China | 26 | 1 | 1.19 | 3 | 0.11-17.98 |  | 0 |  |  | |  |  |
| Ghana | 26 | 3 | 0.31-0.36 | 1 | 0.17-0.36 |  | 0 |  |  |  | |  |
| India | 20 | 0 |  | 0 |  |  | 4 | 0 |  | 0 | |  |
| Italy | 18 | 8 | 0.16-1.00 | 2 | 0.10-0.38 |  | 0 |  |  |  | |  |
| Nigeria | 23 | 16 | 0.19-13.85 | 4 | 0.18-6.01 |  | 0 |  |  |  | |  |
| Paraguay | 21 | 1 | 0.3 | 1 | 0.16 |  | 4 | 0 |  | 0 | |  |
| Serbia | 12 | 12 | 0.78-17.26 | 8 | 0.24-8.07 |  | 0 |  |  |  | |  |
| South Africa | 25 | 17 | 0.25-7.04 | 8 | 0.12-1.43 |  | 0 |  |  |  | |  |
| USA | 14 | 0 |  | 1 | 0.12-0.28 |  | 15 | 15 | 0.37-274.52 | 10 | | 0.46-114.25 |
|  |  |  |  |  |  |  |  |  |  |  | |  |

The lowest values in the concentration range were ≥ LODs (atropine = 0.03µg kg^-1^ and scopolamine = 0.08 µg kg^-1^).

Supplementary Table 3. Soy samples from different regions globally contained atropine plus scopolamine beyond the permitted level of 1 µg kg^-1^.

| Sample origin | atropine + scopolamine concentration in soybeans (µg kg^-1^) | | |  | Sample origin | atropine + scopolamine concentration in soymeal, (µg kg^-1^) | | |
| --- | --- | --- | --- | --- | --- | --- | --- | --- |
|  | Atropine | Scopolamine | Atropine + Scopolamine |  |  | Atropine | Scopolamine | Atropine + Scopolamine |
| Canada | 63.82 | 18.33 | 82.15 |  | Argentina | <LOD | 1.24 | 1.24 |
| Canada | 70.11 | 12.82 | 82.93 |  | Argentina | <LOD | 2.17 | 2.17 |
| China | 1.19 | 0.53 | 1.72 |  | Argentina | <LOD | 3.32 | 3.32 |
| China | <LOD | 17.98 | 17.98 |  | Argentina | 0.75 | 33.49 | 34.24 |
| Italy | 1 | 0.38 | 1.38 |  | Argentina | 1.81 | 141.38 | 143.19 |
| Nigeria | 1.06 | <LOD | 1.06 |  | Brazil | <LOD | 4.35 | 4.35 |
| Nigeria | 1.8 | <LOD | 1.8 |  | Brazil/Paraguay | <LOD | 1.1 | 1.1 |
| Nigeria | 2.01 | <LOD | 2.01 |  | Canada | 1.62 | 0.41 | 2.03 |
| Nigeria | 2.82 | 0.47 | 3.29 |  | USA | 1.12 | <LOD | 1.12 |
| Nigeria | 3.68 | 0.57 | 4.24 |  | USA | 1.01 | 0.46 | 1.47 |
| Nigeria | 8.83 | 1.07 | 9.9 |  | USA | 0.37 | 2.84 | 3.21 |
| Nigeria | 13.85 | 6.01 | 19.86 |  | USA | 0.38 | 3.05 | 3.44 |
| South Africa | 1.2 | <LOD | 1.2 |  | USA | 5.77 | 2.67 | 8.45 |
| South Africa | 1 | 0.32 | 1.31 |  | USA | 7.33 | 2.05 | 9.38 |
| South Africa | 0.66 | 0.87 | 1.53 |  | USA | 10.82 | 3.24 | 14.06 |
| South Africa | 1.18 | 0.45 | 1.63 |  | USA | 11.06 | 3.54 | 14.6 |
| South Africa | 1.39 | 0.57 | 1.96 |  | USA | 11.23 | 3.81 | 15.04 |
| South Africa | 7.04 | 1.43 | 8.47 |  | USA | 51.55 | 13.33 | 64.89 |
|  |  |  |  |  | USA | 274.52 | 114.25 | 388.77 |

LOD: atropine = 0.03µg kg^-1^ and scopolamine = 0.08 µg kg^-1^

LOQ: atropine= 0.10 µg kg^-1^ and scopolamine= 0.25 µg kg^-1^

Supplementary Figure 1. Chromatographic separation of atropine and scopolamine (0.1 ppb spiked in blank soybean extract) using a Phenomenex Gemini C18 column with H_2_O (mobile phase A) and methanol (mobile phase B) with 0.1% formic acid.


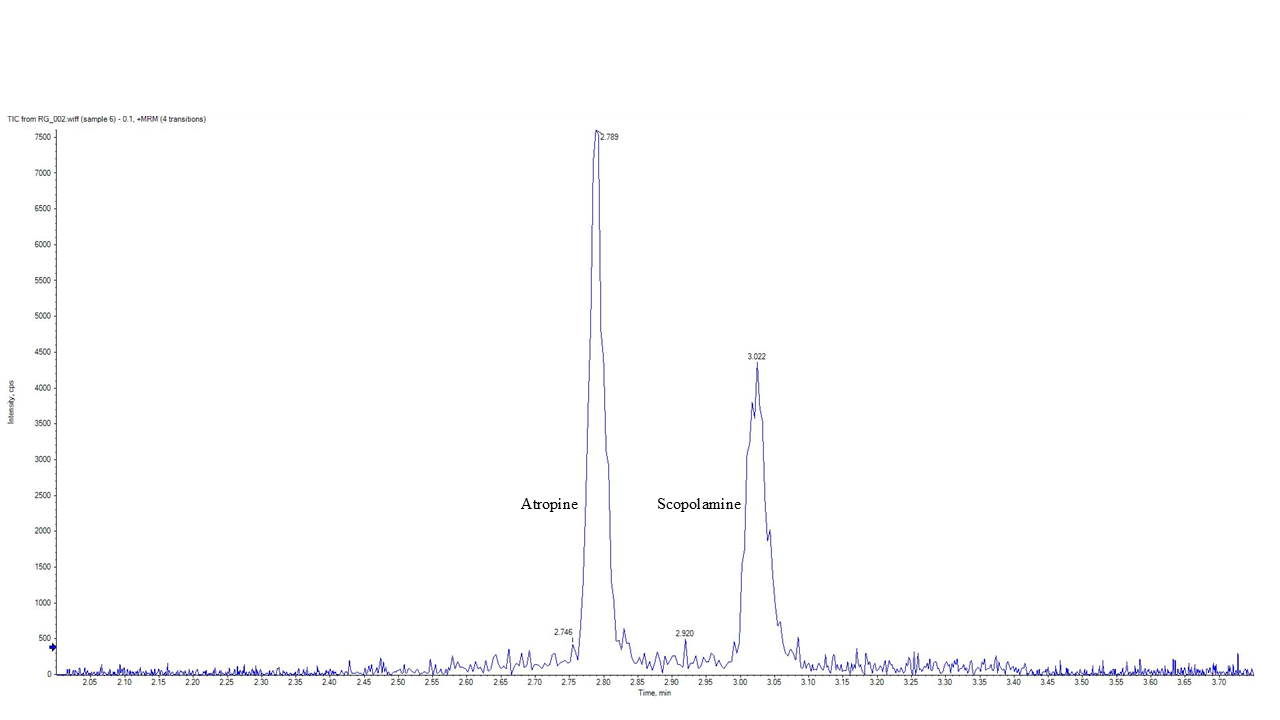

Supplement: Supplementary file 1 — Supplementary file1 (DOCX 141 kb) [file 12403_2025_718_MOESM1_ESM.docx]
